# Chronic Obstructive Pulmonary Disease and Non-Small Cell Lung Cancer an association

**DOI:** 10.7150/jca.90594

**Published:** 2024-01-01

**Authors:** Paul Zarogoulidis, Rena Oikonomidou, Dimitris Petridis, Haidong Huang, Chong Bai, Eleni-Isidora perdokouri, Anastasios Vagionas, Wolfgang Hohenforst-Schmidt, Christoforos Kosmidis, Konstantinos Sapalidis, Panagoula Oikonomou, Christina Nikolaou, Charalampos Charalampidis, Dimitrios Matthaios, Athanasia Pataka, Chrysanthi Sardeli

**Affiliations:** 1 3 rd University General Hospital, ``AHEPA`` University Hospital, Thessaloniki, Greece.; 2 Pulmonary Department, General Clinic Euromedica, Thessaloniki, Greece.; 3 Health Center of Evosmos, Thessaloniki, Greece.; 4 Department of Food Science and Technology International Hellenic University, Alexander Campus, Sindos, Thessaloniki.; 5 Department of Respiratory & Critical Care Medicine, Changhai Hospital, the Second Military Medical University, Shanghai, P. R. China.; 6 Oncology Deprtment, General Hospital of Volos, Greece.; 7 Oncology Department (NHS), General Hospital of Kavala, Kavala, Greece.; 8 Sana Clinic Group Franken, Department of Cardiology / Pulmonology / Intensive Care / Nephrology, "Hof" Clinics, University of Erlangen, Hof, Germany.; 9 Second Department of Surgery, University Hospital of Alexandroupolis, Medical School, Democritus University of Thrace, Alexandroupolis, Greece.; 10 Pathology Department, University of Cyprus, Cyprus.; 11 Oncology Department, General Hospital of Rhodos, Rhodos, Greece.; 12 Pulmonary Department, G.Papnikolaou General Hospital, Aristotle University of Thessaloniki, Greece.; 13 Department of Pharmacology & Clinical Pharmacology, School of Medicine, Aristotle University of Thessaloniki, Thessaloniki, Greece.

**Keywords:** COPD, Nutrition, NSCLC, spirometry, DLCO

## Abstract

**Objectives:** Lung cancer is known to be associated with chronic obstructive pulmonary disease. Moreover; nutritional status is associated with chronic obstructive disease treatment and lung cancer. Our aim was to evaluate the interaction of the COPD status and treatment of non-small cell lung cancer.

**Methods:** Eighty-two patients were enrolled in our multicenter study. Chronic obstructive disease stage, spirometry and treatment was recorded along with the treatment and Body Mass Index (BMI), Mediterranian Diet Score, Pack Years, Basic Metabolsim (RMR) (kcal/day), VO₂ (ml/min), Ve (lt/min) and Physical Activity. The statistical analysis was performed using the JMP 14.3 (SAS Inc 2018) software.

**Results:** The drug pairs showed a steady and unchanged by time health condition for 48 patients. Overall, 31 patients were recorded with worse COPD health conditions. The one-way ANOVA clearly indicated that chemotherapy induced the best FEV_1_-difference conditions with a positive effect of 8.56 mean FEV volume, the combined treatment simply did not have an effect (-0.9), while immunotherapy and patients receiving radiation decreased their FEV_1_ volume down to -4.23 and -5.15 mean values.

**Conclusions:** Patients receiving chemotherapy alone had their chronic obstructive disease improved with less drugs and exacerbations, while patients receiving immunotherapy had their chronic obstructive disease stable, while all other treatment combinations worsened the patients chronic obstructive disease. Nutritional status did not affect the chronic obstructive disease of these patients in any way.

## Introduction

Lung cancer is the leading cause of death among cancer patients. The association between smoking habit and lung cancer has been well established several years ago 1. Unfortunately we do not have blood tests for early lung cancer detection as we have with prostate cancer, gastrointestinal cancer and obstetrics cancer. Therefore lung cancer is diagnosed at a late stage inoperable disease 2, 3. We have novel tools for lung cancer diagnosis and staging, however; systematic treatment is usually administered in most patients 4. Several minimal invasive techniques such radial endobronchial ultrasound (EBUS), convex probe and computed tomography (CT) guided biopsies are used in the everyday clinical practice 5, 6. Positron emission computed tomography (PET-CT) along with convex probe endobronchial ultrasound is used for lung cancer staging. Most of these patients have already been diagnosed with chronic obstructive pulmonary disease (COPD) or their COPD is diagnosed upon lung cancer diagnosis 7. Careful care should be taken when ventilating these patients during the interventional procedures and Jet-Ventilation is the preferable mode 8. In our study we included non-small cell lung cancer (NSCLC) patients. Currently we have several treatments for non-operable non-small cell lung cancer (NSCLC) patients with chemotherapy, radiotherapy, tyrosine kinase inhibitors, immunotherapy or combinations 4, 9-11. After we acquire biopsy we investigate for the following gene expressions epidermal growth factor receptor (EGFR) or T790M, anaplastic lymphoma kinase (ALK), Programmed death-ligand 1 (*PD*-*L1*), Proto-oncogene tyrosine-protein kinase (ROS-1), proto-oncogene encodes and proto-oncogene (B-Raf) 10, 12. Moreover; it has been observed that the nutritional status and metabolic rate of COPD patients are affected by their disease. It has been observed that high protein concentration in the everyday nutrition reduces COPD exacerbations 13. Moreover; the nutritional status of lung cancer patients has been closely associated with treatment efficiency and survival 14-18. In our current study we evaluated the chronic obstructive disease of the patients along with their cancer treatment and their nutritional status and metabolic rate, in an effort to identify how these three parameters interact.

## Patients and Methods

In our retrospective study we included eighty-two non-small cell lung cancer (NSCLC) patients with a diagnosed chronic obstructive pulmonary disease (COPD). All patients were stage IV Non-Small Cell Lung Cancer patients and received intravenous treatment with or without additional radiotherapy. Thirty patients Department of Respiratory & Critical Care Medicine, Changhai Hospital, the Second Military Medical University, Shanghai, P. R. China. 37 Pulmonary Department, General Clinic Euromedica, Thessaloniki, Greece, and 15 Sana Clinic Group Franken, Department of Cardiology / Pulmonology / Intensive Care / Nephrology, "Hof" Clinics, University of Erlangen, Hof, Germany. The diagnosis of lung cancer was made with biopsy (different types of methods were used according to the patient`s computed tomography findings in the thorax). Moreover; positron emission computed tomography was performed to each patient in order to identify the disease stage according to current guidelines 19. All patients were 18 years old and above and they were fit to receive any kind of treatment for their disease. All patients had ECOG status 0-2 upon inclusion. The study was approved by the investigational review board our department (3^rd^ University General Hospital, ``AHEPA`` University Hospital, Thessaloniki, Greece, which belongs to the institution Aristotle University of Thessaloniki protocol approval number 23/21). An informed consent was obtained by the authors from all patients. The study was carried out according to the Helsinki Declaration for the participation of humans and good practice. Thirty-five patients had squamous cell carcinoma and 47 adenocarcinoma. All patients were Stage IV. Moreover; we conducted to all patients next generation sequencing (NGS) and none of the patients had EGFR, ALK, MET, ROS1 or BRAF mutation. For example when PD-L1 was ≥50% we administered pembrolizumab, if less ≤50% then combination of chemotherapy plus immunotherapy was administered. The main chemotherapy drugs were carboplatin with permetrexed, or carboplatin with nab-paclitaxel or paclitaxel. Radiotherapy was also administered were appropriate. All patients had first line treatment and we stopped recording any data upon death or after completion of six cycles of therapy.

Their chronic obstructive disease (COPD) stage was recorded upon their first treatment along with their medication and a spirometry was performed 20. COPD and lung cancer are two separate diseases. The main aim of the study was to evaluate any association between lung cancer treatment and COPD status plus nutritional status. Evaluation of their COPD was performed after the first line completion which differed from 180 days based on their treatment with spirometry. Regarding the spirometry values we included only the forced expiratory volume in 1 second (FEV_1_) as this is the most important value of spirometry and easily recorded in many centers even not pulmonary departments. We divided COPD as follows in our excel file: 1) mild, 2) moderate, 3) severe and 4) very severe according the last COPD guidelines 20. Moreover; treatment for each patient was recorded as follows: 1) two drugs inhaled bronchodilator plus inhaled cortisone (aerosol or powder), 2) three drugs inhaled bronchodilator plus inhaled cortisone (aerosol or powder) plus ipratropium, 3) nebulizer with two drugs inhaled bronchodilator plus inhaled cortisone and 4) nebulizer with two drugs inhaled bronchodilator plus inhaled cortisone plus ipratropium. Regarding their lung cancer, the following combinations were administered: 1) chemotherapy alone, 2) chemotherapy and immunotherapy, 3) radiotherapy plus chemotherapy, 4) radiotherapy plus immunotherapy). The above values can be used to interpret the statistical analysis in the following section. The following parameters were recorded upon diagnosis and last follow-up (first cycle completion), measuring at that interval the weight, fat, lean and body mass index, relative metabolic rate RMR, physical acitvity and breathe index (VO₂).

### Statistical analysis

#### Treatment interaction

The treatment is questioned on how it could influence the change of forced expiratory value (FEV_1_) between the initial and last therapy cycle (FEV_1_-difference), the survival time and the corresponding number of COPD drug shifts at the same period.

The number of administered drugs was concatenated in shift pairs from initial to final phase forming two digits, e.g. the level 12 denotes a transition from one administered drug to two of those. The digit 3 denotes the unique administration of nebulizer and the digit 4 the same drug plus 3 more. Therapy means against FEV_1_-difference and survival time were examined for potential effects by employing the one-way Analysis OF Variance (ANOVA) and a single correspondence analysis between drugs shift levels and therapy treatment was conducted. The statistical analysis was performed using the JMP 14.3 (SAS Inc 2018) software.

##### Biomarkers

Biomarkers such as BMI, % predictive Harris Benedict equation, body fat, lean and dry lean body mass and maximum rate of oxygen consumption (VO_2_) were examined as initial minus final response and as potentially affected by the COPD drug pair shifts and FEV_1_-diff. Mediterranian diet score and pack years were also considered in the analysis.

## Results

**Table 1** presents the frequency distribution of patients in the various therapy treatments and COPD initial and final therapy administrations. Patients were mainly treated with radiation+ (39%) and less with immunotherapy (13.4%). The initial administration of one or two drugs included 75.6% of patients while nebulizer was reduced to only 4 individuals. The administration of one drug in the final phase was reduced to 50% (14 patients), that of 2 and 4 drugs remained stable but nebulizer increased 4-fold including 17 patients.

The drug shift pairs showed a steady and unchanged by time health condition for 48 patients (11, 22, 33, 44), that is 58.6% of the total sample (**Table 2**), a shift by one more drug for levels 12 (17.1%), 23 (14.6%) and 34 (2.4%) and surprisingly an inverse shift 43 for 3 patients (3.7%). Overall, 31 patients were recorded with worse COPD health conditions.

**Figure 1:**
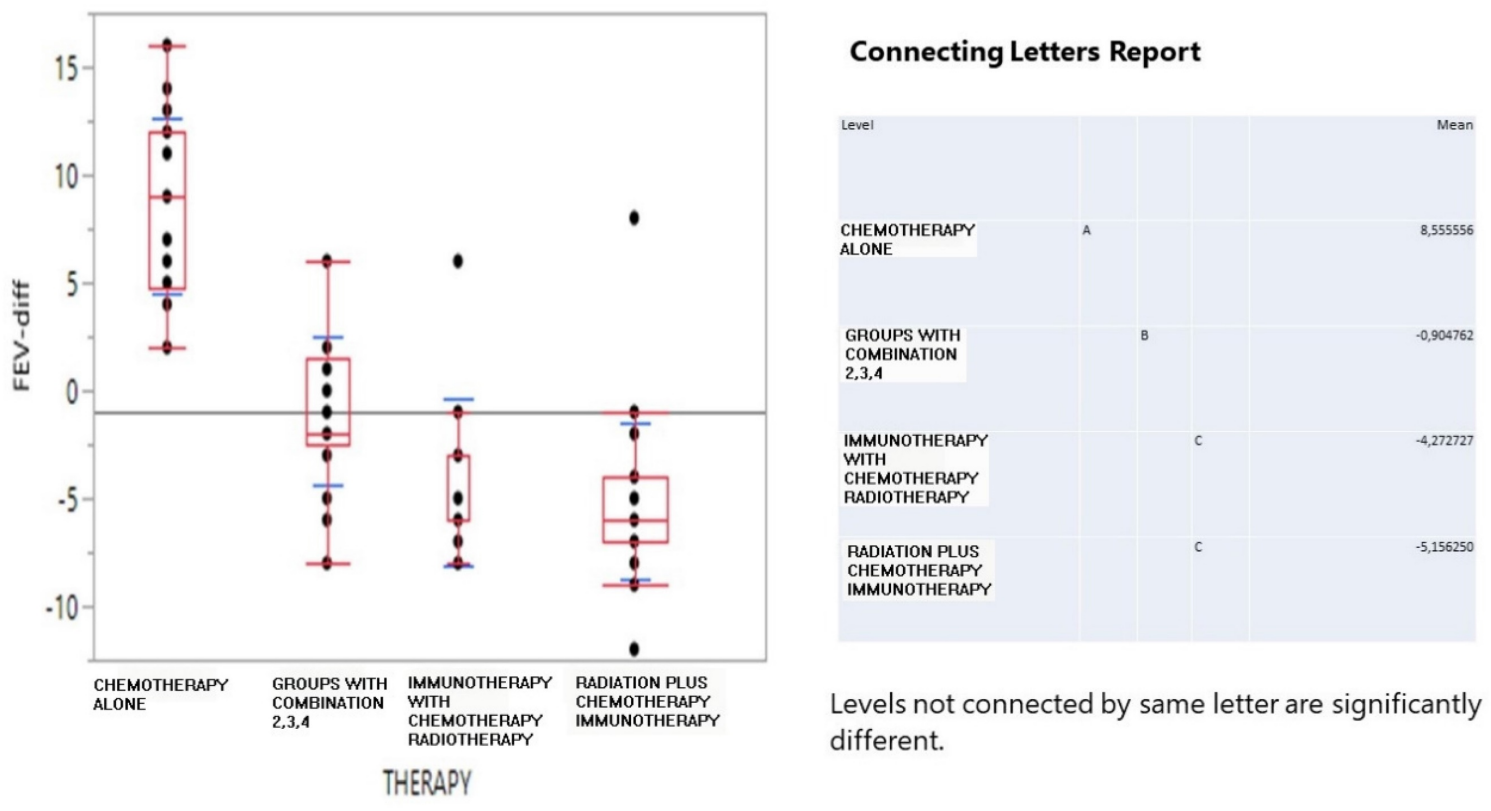
Interval boxplot of survival days (in log_10_ transformation) according to therapy scheme and a pairwise comparison of means based on the Student-Newman-Keuls test.

The one-way ANOVA in **Figure 1** clearly indicated that chemotherapy induced the best FEV_1_-difference conditions with a positive effect of 8.56 mean FEV volume, the combined treatment simply did not have an effect (-0.9), while immunotherapy and radiation+ decreased the FEV_1_ volume down to -4.23 and -5.15 mean values.

**Figure 2:**
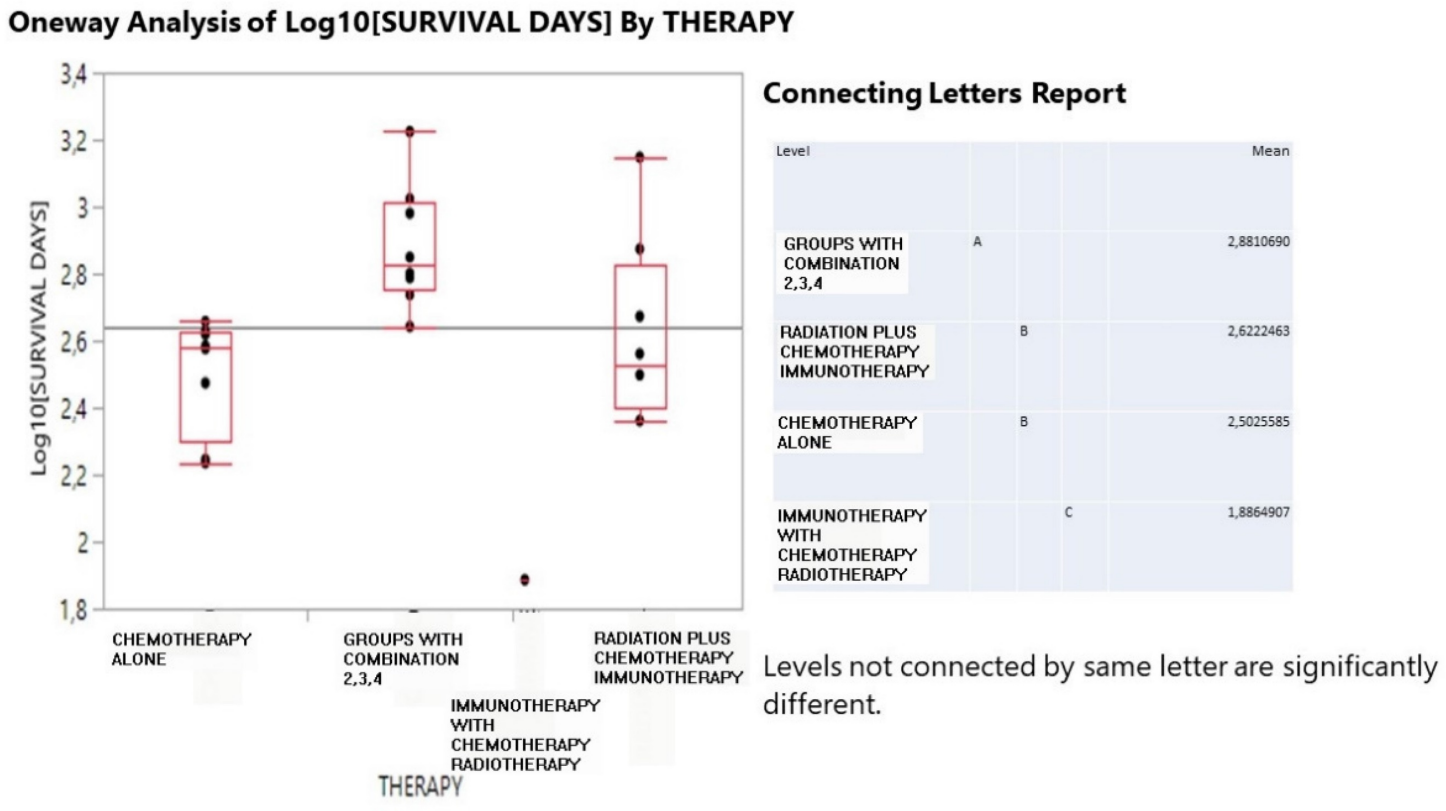
Interval boxplot of survival days (in log_10_ transformation) according to therapy scheme and a pairwise comparison of means based on the Student-Newman-Keuls test.

On the other hand, in **Figure 2**, survival time (in log_10_ transformation) was best achieved by the combined treatment (760 days), moderately by radiation+ (419) and chemotherapy (318) and least by immunotherapy (77 days, although it should be thought with caution as limited to one patient record).

**Figure 3:**
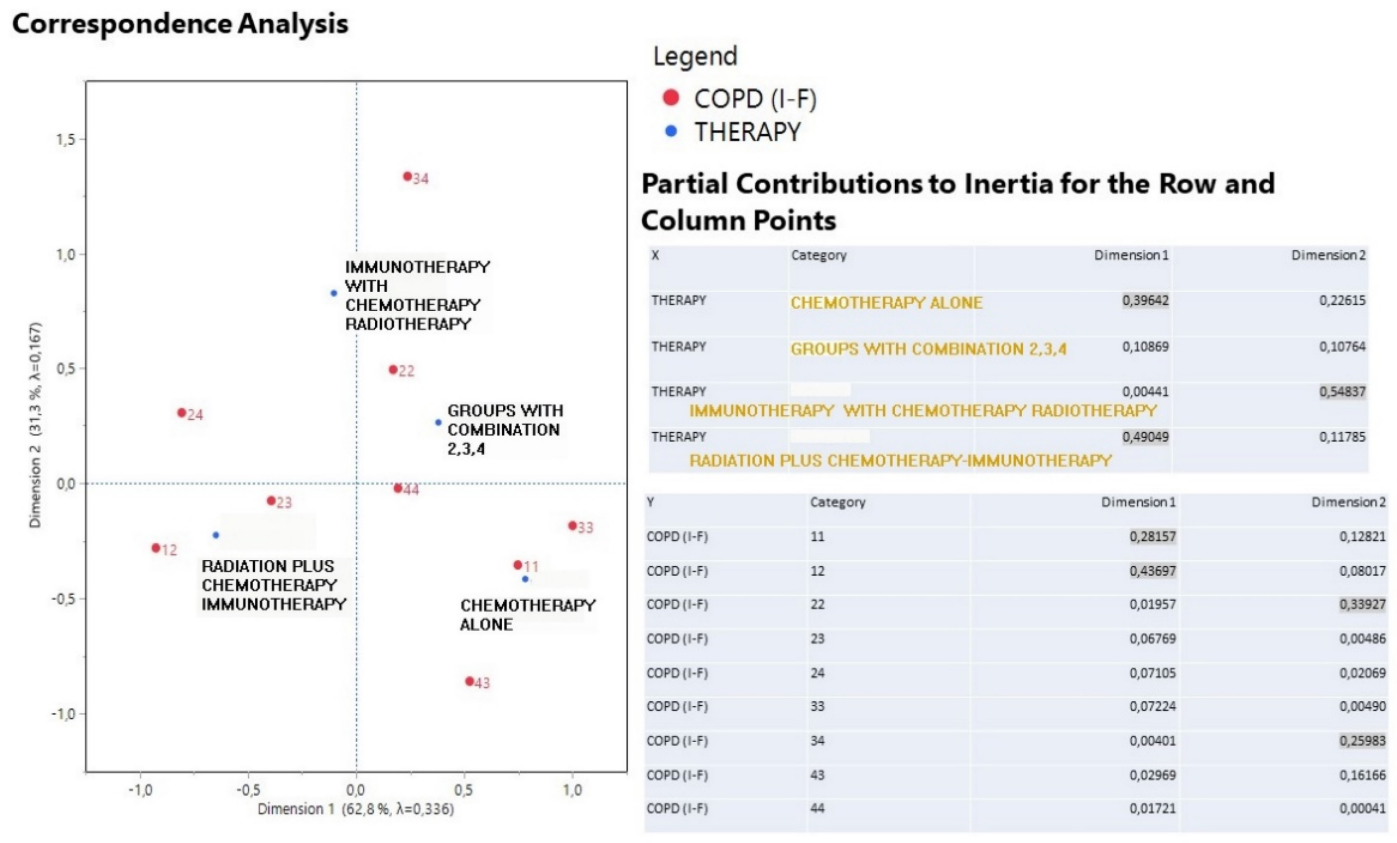
A correspondence analysis output including the first two dimensions plot of therapy scheme and drug shift pairs and the partial contribution to inertia of both variable categories.

**Figure 3** describes the correspondence analysis between therapy treatment and drug shift pairs administration in joint with the partial contribution to the inertia of the variable categories. High coordinates signal for a significant effect of a particular treatment and a drug shift pair. Points in the graph positioned close each other form clusters with specific information. Dimension 1 is best described by chemotherapy and radiation+ (39.6% and 49.0% contribution) but in an opposite direction in the graph and by drug shift pairs 11 and 12 (28.2% and 43.7%). Chemotherapy is also indicative of the surrounding drug shift pairs 11, 33 and radiation+ informs for the presence of drug shifts 12, 23 and 24. In fact, chemotherapy reflects a stable COPD health condition for one drug administration and solely nebulizer and even an improved condition due to a drug shift from 4 drugs to only nebulizer. On the contrary, radiation+ is affiliated with worse COPD conditions because here transitions happen from 1 to 2 drugs and from 2 to 3 drugs and also a double jump from 2 to 4 drugs. Immunotherapy strongly describes the dimension 2 (54.8% contribution) in conjunction with drug shift pair 34 (26%) and 22 (33.9%). It turns out that immunotherapy indicates a switching form one drug (nebulizer) to additionally 3 drugs, whereas the combined treatment is characterized by the stable drug shift pairs 22 and 44.

Biomarkers were tested with t-test and one-way ANOVA on how potentially have been influenced by the FEV_1_-diff and COPD conditions (**Table 3**). FEV_1_-difference was reformed to positive and negative values and COPD drug pair shifts to stable (11, 22, 33, 44), positive (43) and negative (12, 23, 34, 24) values. A sole statistically significant effect was recorded and that for BMI (p=0.042) which manifested an improvement in value at the negative FEV_1_ phase (0.30>-0.69).

In summary, FEV_1_ improved only for patients under chemotherapy patients, those with the highest survival time with combined therapy. Moreover; those with stable COPD under chemotherapy and secondly in patients under with combination therapy. No particular effects of FEV_1_ and COPD conditions on biomarkers were documented in the study.

## Discussion

Chronic obstructive disease is closely associated with tobacco use and in some cases with the working environment. Moreover; the populated environment play a crucial role in several regions. It is usually diagnosed from the age of 40 and above. COPD has been associated with lung cancer for many years 1,6. Different types of non-small cell lung cancer receive different treatment. The nutritional status of the patient has been associated with treatment efficiency and survival in previous studies. Tyrosine kinase inhibitors and immunotherapy are known to induce pneumonitis. Immunotherapy can also induce pericarditis and pleura effusion 8.

Radiation can induce also damage to the lung parenchyma although we have new radiation equipment and methodology. Therefore these patients which in many cases their COPD is underdiagnosed should be closely followed with pulmonary function tests. In our study we performed only spirometry as it is an easy to perform examination from many physicians 12.

Major limitation of our study is that we did not perform diffusing capacity of lung for carbon monoxide (DLCO), because not all of the collaborating departments had this equipment. In the current study we wanted to evaluate which treatment affected the COPD status and we observed that patients receiving only chemotherapy had their FEV_1_ improved, they remained in the same stage, however; their medication for several of this patients changed by reducing dosages than upon initiation of cancer treatment. Another limitation of our study was the small number of patients included receiving immunotherapy. Also, the small number of patients included in the study. Treatment combination chemotherapy with/or immunotherapy simply did not have an effect (-0.9), while immunotherapy and radiation decreased the FEV_1_ volume down to -4.23 and -5.15 mean values. The following parameters were recorded upon diagnosis and last follow-up (first cycle completion), measuring at that interval the weight, fat, lean and body mass index, relative metabolic rate RMR and breath index (VO₂). After the proper combinations with FEV_1_ and COPD stage correlation, their changes did not have any effect on the COPD status, chemotherapy alone is an independent factor for COPD. This observation can be explained because chemotherapy is known to have a fast effect in bulky disease in comparison to other treatments.

However; prolonged survival was observed with combination treatments. Although nutritional status plays a significant role in survival for lung cancer patients, the parameters of the metabolic rate that we included were not statistically correlated with COPD, FEV_1_ and survival. Further studies are needed to verify our findings 12,13.

## Conclusion

FEV_1_ improvement is uniquely achieved by chemotherapy, highest survival time by the combined therapy (chemotherapy and immunotherapy, radiotherapy plus chemotherapy and radiotherapy plus immunotherapy) stable COPD health conditions by chemotherapy and secondly by combined therapy and worse COPD conditions by radiation and immunotherapy at the higher levels of drug administration. No particular effects of FEV_1_ and COPD conditions on biomarkers were documented in the study. Metabolic rate did not affect the chronic obstructive disease of these patients and their survival in any way. There was no association between BMI and COPD exacerbations, nor BMI and survival.

## Figures and Tables

**Table 1:** Numerical and percentage patient distribution according to therapy scheme and number of COPD drugs administration in the beginning and end of the study.

THERAPY			
CHEMOTHERAPY	COMBINATION	IMMUNE	RADIATION+
			
18	21	11	32
22,00%	25,60%	13,40%	39,00%
			
COPD-initial patients and number of drugs			
1	2	3	4
28	34	4	16
34,10%	41,50%	4,90%	19,50%
			
			
COPD-final patients and number of drugs			
1	2	3	4
14	33	17	18
17,10%	40,20%	20,70%	22,00%

**Table 2:** Numerical and percentage distribution of the COPD drug shift pairs.

Level	N	Proportion
11	14	0,171
12	14	0,171
22	19	0,232
23	12	0,146
24	3	0,037
33	2	0,024
34	2	0,024
43	3	0,037
44	13	0,159
Total	82	1

**Table 3:** Cross tabulation of mean biomarkers according to FEV_1_-diff positive and negative effects and to COPD drug pair shifts according to positive, negative and stable health conditions. Asterisk denotes a significant effect (p=0.042).

		BMI I-F (kg/m²)	%Pred (Harris Benedict) I-F	FAT I-F (%)	LEAN I-F (kg)	DRY LEAN I-F (kg)	VO₂ I-F (ml/min)	MEDITERRANIAN DIET SCORE	PACK YEARS
FEV_1_ diff	n								
negative	54	0,30^*^	-5,67	0,57	-0,17	0,30	17,56	30,78	82,93
positive	27	-0,69	-11,44	1,05	-2,02	-0,07	23,70	30,67	85,73
									
COPD	n								
negative	31	0,02	-5,94	0,70	-0,45	0,71	18,97	31,48	72,27
positive	3	0,20	-5,00	3,23	-3,63	0,30	25,33	28,00	91,60
stable	48	-0,09	-8,65	0,52	-0,80	-0,34	19,25	30,56	89,11
